# Persistence of Asbestos-Containing Friction Materials in the Hungarian Waste Stream Twenty Years After the European Union Ban

**DOI:** 10.3390/ijerph23060802

**Published:** 2026-06-16

**Authors:** Áron Szandi, Zsombor Balog, Krisztián Sándor Zaka, Gergely Zoltán Macher

**Affiliations:** 1 Department of Applied Sustainability, Albert Kázmér Faculty of Agricultural and Food Sciences, Széchenyi István University, Egyetem tér 1, H-9026 Győr, Hungary; 2 Sustainability Competence Centre, Széchenyi István University, Egyetem tér 1, H-9026 Győr, Hungary

**Keywords:** asbestos-containing friction materials, hazardous waste, occupational exposure, waste management, environmental health

## Abstract

**Highlights:**

**Public health relevance—How does this work relate to a public health issue?**

- Asbestos-containing brake and clutch materials are still present in hazardous waste streams in Hungary nearly two decades after the EU ban.
- Waste handling, dismantling, and disposal activities may represent ongoing occupational exposure pathways.

**Public health significance—Why is this work of significance to public health?**

- The study provides national-level evidence on the persistence and distribution of a legacy carcinogenic material in the waste management system.
- Findings reveal potential gaps in identification, reporting, and long-term risk control of asbestos-containing components.

**Public health implications—What are the key implications or messages for practitioners, policy makers and/or researchers in public health?**

- Strengthened monitoring, improved classification practices, and targeted occupational safety measures are needed in vehicle dismantling and hazardous waste sectors.
- Policy integration between circular economy strategies and asbestos risk management is essential to prevent residual exposure.

**Abstract:**

Although asbestos has been banned in the European Union since 2005, asbestos-containing materials, such as brake pads and clutch linings, may still occur in waste streams due to the long service life of vehicles, legacy equipment, and international trade in spare parts. The persistence of these materials raises environmental and occupational health concerns, particularly in waste management systems. This study aims to assess the presence, temporal trends, and sectoral distribution of asbestos-containing friction materials in the Hungarian waste management system two decades after the EU ban, and to evaluate the associated regulatory and occupational risk implications. The analysis is based on national hazardous waste datasets classified under EWC code 16 01 11* (asbestos-containing brake pads), with a specific focus on this waste category rather than on the full range of asbestos-related waste streams recorded in the national database. The results indicate that asbestos-containing friction materials are still present in the waste stream, with measurable quantities recorded annually. Despite regulatory control, identification challenges and potential misclassification may contribute to underreporting. The continued occurrence of asbestos-containing materials highlights the persistence of legacy hazardous materials within circular economy systems. Strengthened monitoring, improved identification protocols, and enhanced occupational safety measures are necessary to mitigate residual exposure risks. The findings underline that asbestos is not merely a historical issue but remains a contemporary environmental and public health challenge.

## 1. Introduction

Asbestos remains one of the most significant occupational and environmental carcinogens worldwide [1,2]. Despite regulatory bans in many jurisdictions, including the European Union since 2005 [3,4], asbestos-related diseases continue to impose a substantial public health burden due to long latency periods and legacy exposure sources [5,6,7]. While the production and marketing of asbestos-containing materials have been prohibited within the EU, residual materials embedded in existing infrastructure, vehicles, and industrial equipment persist in technical systems and waste streams [1,8]. Among these legacy products, asbestos-containing friction materials (abbreviation: ACFMs) such as brake pads and clutch linings represent a specific but underexamined exposure pathway [9,10]. Historically, chrysotile asbestos was widely used in automotive and industrial friction components because of its thermal resistance and durability [11,12]. Although new asbestos-containing products are banned, components manufactured before regulatory restrictions, imported spare parts, and long service-life vehicles may continue to enter dismantling and waste management systems decades after prohibition [13].

Waste management systems constitute a critical interface between environmental protection and occupational health [14,15]. Workers engaged in vehicle dismantling, maintenance, hazardous waste handling, and landfill operations may encounter ACFMs during mechanical processing, fragmentation, and disposal [2,13,16]. In this context, the potentially exposed working population primarily includes vehicle dismantling workers, automotive maintenance personnel, hazardous waste handlers, transport and storage workers, recycling operators, and landfill personnel whose tasks may involve the removal, sorting, packaging, transfer, mechanical disturbance, or final disposal of asbestos-containing friction components. Even when classified as hazardous waste under European Waste Catalogue (abbreviation: EWC) code 16 01 11*, effective risk control depends on accurate identification, reporting, segregation, and regulatory compliance [17,18]. Misclassification, incomplete documentation, or insufficient awareness may lead to residual occupational exposure [1]. Because waste statistics are reported by waste category and economic activity rather than by individual workers, they do not allow a direct estimate of the number of exposed workers; however, they can indicate the sectors and activities in which residual occupational exposure is most likely to occur. Despite the recognized carcinogenicity of asbestos and the extensive literature on asbestos-related diseases, limited empirical research has examined the persistence of ACFMs within post-ban waste streams at the national scale [8,19]. Most studies focus on exposure in construction, demolition, or historical industrial settings, while the waste management dimension remains comparatively underexplored [20,21,22]. As circular economy strategies promote material recovery, reuse, and extended product lifecycles [23], legacy hazardous components may re-emerge as hidden risk factors within recycling-oriented systems [24,25]. Hungary provides a relevant case study due to its vehicle fleet structure, industrial legacy, and regulatory alignment with EU asbestos legislation. However, systematic analysis of national hazardous waste data to assess the ongoing presence and distribution of ACFMs has not yet been comprehensively conducted. Understanding whether such materials persist in measurable quantities, and in which sectors they concentrate, is essential for evaluating long-term regulatory effectiveness and occupational risk management [9,16,26].

Accordingly, this study aims to assess the occurrence, temporal trends, and sectoral distribution of ACFMs within the Hungarian waste management system nearly two decades after the EU ban. The study addresses the following research questions:

- RQ1: Do ACFMs continue to appear in the Hungarian hazardous waste stream twenty years after the EU ban?
- RQ2: What temporal trends characterize their generation patterns?
- RQ3: Which economic sectors are primarily responsible for their occurrence?
- RQ4: Do current classification and reporting practices adequately reflect potential exposure risks?

Based on regulatory expectations and legacy material dynamics, the following hypotheses are formulated:

**H1.**  *ACFMs remain present in measurable quantities within the hazardous waste stream despite the EU ban.*

**H2.**  *Waste generation patterns are sectorally concentrated in vehicle dismantling and maintenance activities.*

**H3.**  *Reporting inconsistencies and classification challenges contribute to potential underestimation of actual occurrence.*

**H4.**  *The persistence of these materials represents a residual occupational health risk within the waste management sector.*

By integrating waste statistics analysis with regulatory and public health perspectives, this study contributes to the understanding of how legacy carcinogenic materials persist within contemporary environmental governance systems. The findings aim to inform occupational safety strategies, regulatory refinement, and evidence-based public health decision-making in the context of long-term asbestos risk management.

## 2. Methodology

### 2.1. Study Design

This study employs a retrospective, nationwide quantitative research design to assess the persistence of ACFMs in the Hungarian waste management system following the European Union asbestos ban. The methodological framework integrates statistical analysis of hazardous waste records with regulatory and occupational health contextual interpretation. The aim is not only to quantify material occurrence but also to evaluate its public health relevance within contemporary waste governance systems.

### 2.2. Data Sources and Study Period

The empirical analysis is based on officially reported Hungarian hazardous waste data obtained from the National Environmental Information System, specifically its Electronic Waste Information System public database. The dataset includes records classified under the European Waste Catalogue (EWC) code 16 01 11*, referring to asbestos-containing brake pads. The study period covers 2006–2023, representing nearly two decades following the implementation of the EU-wide asbestos prohibition. Reporting under this classification is mandatory according to national and EU waste legislation, ensuring standardized categorization and traceability. The analyzed variables include annual generated quantities (expressed in tons), reporting entity sector classification, regional identifiers where available, and declared waste treatment routes. All reported entries under EWC code 16 01 11* within the defined period were included in the analysis. Records lacking quantitative information or identified as duplicates were excluded to ensure internal consistency. Non-asbestos friction materials classified under EWC 16 01 12 were not included in the analysis.

### 2.3. Temporal Trend Analysis

Temporal dynamics were examined through time-series analysis of annual waste generation quantities. Absolute values were evaluated alongside relative year-to-year variation in order to identify fluctuation patterns, structural shifts, and longer-term tendencies. Linear regression modelling was applied to assess the direction and statistical significance of observed trends over time. This analytical approach allows the evaluation of whether the reported occurrence of ACFMs demonstrates decline, stability, or persistence in the post-ban period.

### 2.4. Sectoral and Spatial Distribution Analysis

Sectoral distribution was assessed by categorizing reporting entities according to national economic activity classifications. Proportional contribution analysis was conducted to determine the relative share of each sector in total annual generation. Particular attention was paid to vehicle dismantling, automotive maintenance, and industrial equipment replacement sectors, as these activities represent potential occupational exposure contexts. Where regional identifiers were available, spatial distribution was analyzed at NUTS regional level, referring to the Nomenclature of Territorial Units for Statistics used by the European Union for regional classification and statistical comparison. Regional aggregation enabled identification of potential industrial clustering effects and geographic concentration of hazardous waste generation.

### 2.5. Data Reliability and Limitations

The study relies on officially reported administrative data, which enhances reliability but does not eliminate potential sources of uncertainty. Possible limitations include misclassification of friction materials, underreporting by smaller operators, inconsistencies in sectoral coding, and the absence of direct fiber concentration measurements. Consequently, the analysis reflects reported occurrence rather than direct exposure measurement. Inferences regarding occupational health risk are derived from waste presence and handling context rather than environmental or biomonitoring data.

## 3. Results

### 3.1. Temporal Trends in the Generation of ACFMs

The temporal evolution of EWC 16 01 11* in Hungary between 2004 and 2023 shows a pronounced long-term decline following the entry into force of the national asbestos ban on 1 January 2005. Over the entire study period, a total of 826,625 kg of asbestos-containing friction waste (abbreviation: ACFW) was collected and pre-treated. A particularly sharp peak occurred in 2004, when approximately 43% of the total twenty-year volume was recorded. This pre-ban surge most likely reflects large-scale disposal and stock clearance immediately before the legal prohibition entered into force. The transition from 2004 to 2005 marks the most substantial year-to-year decline in the dataset, exceeding 86%. After 2005, annual quantities remained far below the pre-ban level, indicating that the ban produced an immediate and lasting contraction in the circulation of ACFMs. When 2004 is excluded, the post-ban period is characterized by much lower and more stable annual volumes, confirming that the pre-ban disposal wave strongly distorts the long-term average. Regression analysis supports this interpretation. The fitted exponential model shows a coefficient of determination of R^2^ = 0.7666, indicating a statistically robust downward trend, while the logarithmic model also yields a negative slope (R^2^ = 0.715).

**Figure 1:**
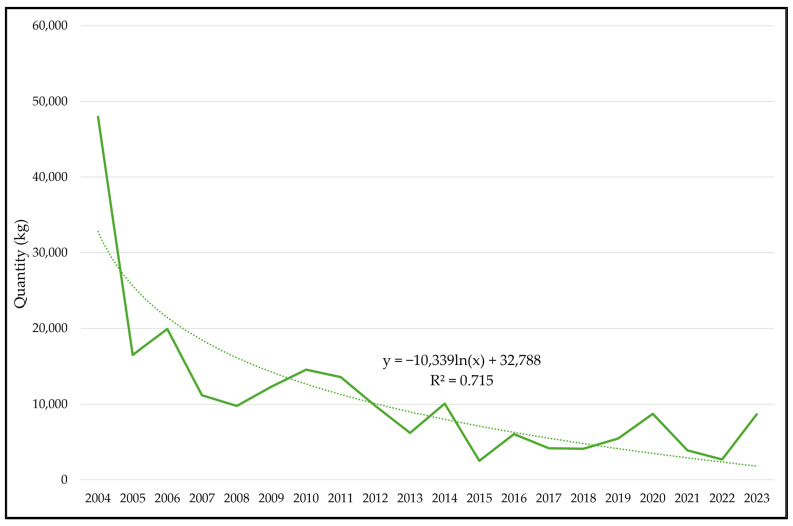
Annual quantity of ACFW in Hungary, 2004–2023 (kg).

Although the overall trajectory is clearly declining, moderate fluctuations remain visible, with secondary peaks around 2009 and 2018. These episodic increases are interpreted as being more plausibly associated with delayed dismantling, inventory clearance, or fleet renewal than with any renewed use of asbestos-containing materials; however, this interpretation is based on the temporal pattern of reported waste quantities rather than on direct operational records from the reporting entities. After 2017, the decline becomes more gradual, suggesting that residual ACFMs persist mainly in long-life technical systems rather than in active product circulation. Annual quantities of ACFMs (EWC 16 01 11*) recorded in Hungary between 2004 and 2023, with a fitted logarithmic trend line. Figure 1 shows a marked decline after the exceptionally high value observed in 2004, followed by lower but still fluctuating annual quantities in the post-ban period. The negative logarithmic trend (R^2^ = 0.715) indicates a persistent long-term decrease, although intermittent peaks suggest delayed dismantling, stock clearance, or the gradual removal of legacy asbestos-containing components from technical systems.

### 3.2. Spatial Distribution of ACFMs

The spatial distribution of ACFW in Hungary between 2004 and 2023 reveals marked territorial asymmetry. Although the national trend is one of long-term decline, the handling of this waste is not evenly distributed across the country. More than three-quarters of the total recorded volume was associated with four regions: Northern Hungary, the Southern Great Plain, Pest, and Budapest. This indicates that both the generation and management of ACFMs remain concentrated within a limited number of economically and infrastructurally significant areas. The most striking pattern concerns treatment centralization. Northern Hungary accounted for 502,510 kg of collected and pre-treated ACFMs over the study period, corresponding to approximately 60.8% of the national total. By contrast, the amount generated locally in the same region was far lower.

This large discrepancy demonstrates that Northern Hungary functions as a national treatment hub rather than merely reflecting local waste production. Similar, though less extreme, imbalances between generated and treated quantities are also observable in Southern Transdanubia and the Southern Great Plain, confirming the importance of inter-regional transport within the hazardous waste management system. Temporal-spatial patterns further indicate that regional peaks were not synchronous. Elevated volumes were recorded in Northern Hungary between 2006 and 2012, in Central Transdanubia around 2014, in the Southern Great Plain around 2020, and in Pest in several later years. These peaks are unlikely to represent renewed asbestos use; rather, they are consistent with delayed dismantling campaigns, industrial restructuring, or the disposal of legacy stocks. Importantly, regions with relatively high generation do not necessarily coincide with regions where treatment activity is concentrated. This territorial dissociation implies that environmental burden and occupational exposure risk are redistributed through waste logistics rather than remaining at the point of origin.

**Figure 2:**
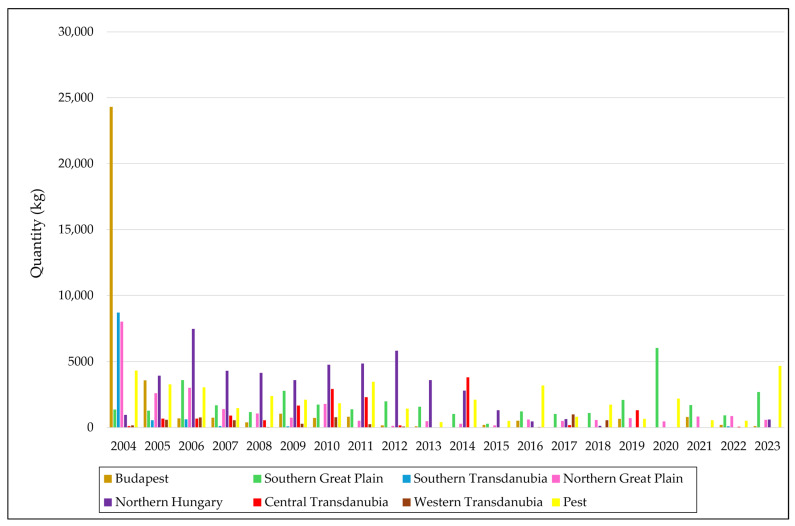
Annual generation of ACFW by region in Hungary, 2004–2023 (kg).

The spatial evidence points to a dual structure: moderate concentration of waste generation in economically active regions and strong concentration of collection and pre-treatment in a small number of infrastructural nodes, especially Northern Hungary. From a public health perspective, this means that declining national quantities do not necessarily imply a uniform reduction in local risk. Instead, exposure potential may remain geographically concentrated in treatment-intensive areas. Regional annual quantities of generated asbestos-containing friction linings (EWC 16 01 11*) in Hungary between 2004 and 2023. Figure 2 shows a pronounced one-off peak in 2004, most notably in Budapest, followed by a sharp decline after the entry into force of the asbestos ban in 2005. In the post-ban period, annual quantities remained substantially lower and displayed uneven regional fluctuations, indicating episodic dismantling, stock clearance, and the delayed removal of legacy asbestos-containing components rather than renewed asbestos use.

### 3.3. Origin Structure of ACFMs

The origin categories of EWC 16 01 11* provide important insight into the institutional pathways through which ACFW continues to circulate in Hungary. Over the 2004–2023 period, the distribution of collected and pre-treated waste by origin category was highly uneven. The dominant share originated from “other producers,” accounting for approximately 88% of the total volume. This category includes industrial operators, commercial maintenance facilities, dismantling enterprises, and other non-household actors, indicating that ACFW is primarily embedded in regulated industrial and commercial flows. By comparison, producer-generated municipal waste represented a much smaller share, waste received from waste management facilities remained limited, and trader-originated quantities were marginal for most of the period. Household contributions were negligible, with only a minimal quantity recorded, and only in 2004.

This is a significant finding because it suggests that ACFMs do not circulate meaningfully through informal residential disposal channels. Instead, they remain overwhelmingly associated with formalized waste management and industrial reporting structures. The temporal development of the origin categories reinforces this interpretation. The “other producers” category dominated the pre-ban disposal peak in 2004 and then declined sharply after 2005, consistent with a one-time clearance of accumulated stock before the legal prohibition. Thereafter, origin-specific volumes stabilized at much lower levels, with only intermittent fluctuations. After 2017, waste received from waste management facilities appears more distinctly as a separate origin category, which may indicate growing specialization within the hazardous waste chain, changes in classification practice, or increasing internal redistribution between facilities. Similarly, the emergence of trader-originated quantities in 2022–2023, although small in absolute terms, may reflect organizational or regulatory changes in the management of residual ACFMs.

### 3.4. Economic-Structural Concentration of ACFMs

The sectoral distribution of ACFW demonstrates that the asbestos legacy in Hungary is not dispersed broadly across the economy, but is concentrated within a narrow set of automotive-related activities. These sectoral results refer specifically to ACFMs recorded under EWC code 16 01 11*, and should therefore not be interpreted as representing the sectoral distribution of all asbestos-containing waste streams in Hungary. Between 2004 and 2023, three economic sectors accounted for approximately 67% of the total recorded ACFM volume: vehicle trade, repair and fuel retail; motor vehicle and motorcycle trade and repair; and the manufacture of road vehicles. This concentration indicates that ACFMs remain structurally embedded in the vehicle life-cycle economy rather than appearing as a generalized cross-sectoral waste stream. The dominance of repair and maintenance-related sectors is particularly important from an occupational health perspective. These activities involve dismantling, abrasion, handling of worn components, and sorting of waste fractions, all of which may create conditions conducive to fiber release. The presence of ACFMs in road vehicle manufacturing should not be interpreted as evidence of ongoing asbestos use in production.

**Figure 3:**
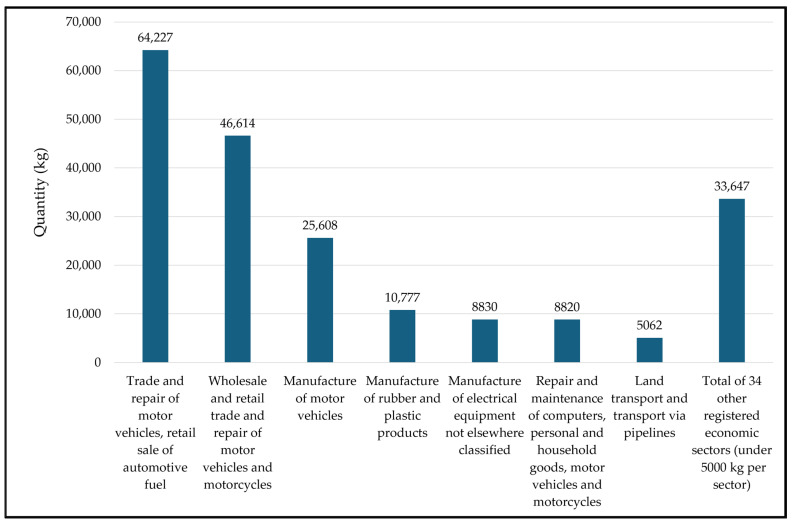
Quantity of ACFW by economic sector in Hungary, 2004–2023 (kg).

More plausibly, it reflects the management of legacy inventories, obsolete spare parts, or asbestos-containing materials encountered during industrial clearance and maintenance operations. In contrast, the absence of substantial quantities in unrelated sectors suggests that ACFMs remain technologically specific. Their persistence is tied to older vehicle fleets, machinery, and associated maintenance systems rather than to the broader structure of the national economy. When considered together with the temporal results, the sectoral pattern points to a lag between regulatory prohibition and actual material disappearance. Although asbestos use was banned, vehicle-related technical systems continue to release residual waste over extended time horizons as older components are repaired, dismantled, and removed from circulation. This sectoral concentration also complements the regional picture described above. Waste generation is economically concentrated in automotive activities, while treatment is geographically concentrated in selected regions. The result is a dual concentration model: economic concentration at the source level and spatial concentration at the treatment level. This combination may intensify occupational and environmental burdens in specific nodes of the waste management system. Cumulative quantities of asbestos-containing friction lining waste (EWC 16 01 11*) by economic sector in Hungary between 2004 and 2023. The largest volumes were recorded in vehicle trade, repair and automotive fuel retail (64,227 kg), followed by wholesale and retail trade and repair of motor vehicles and motorcycles (46,614 kg), and the manufacture of motor vehicles (25,608 kg). Together, these sectors indicate a strong concentration of ACFW within automotive-related activities, while the remaining sectors contributed substantially smaller amounts. This pattern suggests that the post-ban persistence of such waste is structurally linked to vehicle maintenance, dismantling, and related industrial operations (Figure 3).

### 3.5. Generation and Pre-Treatment Dynamics

A comparison between generated quantities and collected/pre-treated volumes reveals that the Hungarian ACFW stream does not operate as a simple annual input-output system. Instead, collected and pre-treated quantities frequently exceed reported generation, indicating that the system is processing not only newly generated waste but also previously accumulated materials and inter-regionally transferred stocks. This persistent imbalance suggests that the hazardous waste stream is shaped by delayed-flow dynamics rather than by immediate one-to-one correspondence between generation and treatment. The discrepancy is particularly informative in the post-ban period. While the legal prohibition sharply reduced the entry of new ACFMs into circulation, treatment activity continued at levels that cannot be explained solely by contemporaneous generation. This implies the ongoing depletion of historical stocks embedded in vehicles, machinery, and storage systems. In addition, the mismatch between generation and treatment may reflect delayed reporting, temporary storage prior to disposal, or classification practices that assign waste more definitively at the treatment stage than at the point of origin.

Temporal decoupling between generation and collection is visible in several years, when local or national peaks in generated quantities do not coincide with peaks in treated volumes. Such asynchrony supports the interpretation of a stock-and-release system, in which waste enters the treatment chain after variable delays. It also aligns with the regional evidence showing strong centralization of treatment activity and frequent movement of hazardous waste across territorial boundaries. From a systems perspective, the Hungarian management of ACFW is best described as a legacy depletion mechanism. The dominant driver of post-ban waste flows is not the introduction of new asbestos-containing materials, but the gradual clearance of older components from the technical infrastructure. This is important for public health because it implies that the highest exposure potential is likely concentrated in handling, sorting, and pre-treatment environments rather than at the original point of waste generation. Even under declining national volumes, treatment-related occupational safeguards therefore remain essential.

### 3.6. Hypothetical Reduction Attributable to Thermal Treatment and Incineration Processes

This subsection presents a conceptual scenario analysis rather than an empirically observed treatment outcome. It is included to illustrate the potential magnitude of mass reduction under assumed asbestos-content scenarios, while the reported Hungarian waste data themselves do not provide direct evidence on actual post-treatment residual masses or mineralogical transformation. The long-term decline in recorded ACFM quantities raises an important interpretive question: does the observed reduction reflect actual destruction of asbestos fibers through thermal treatment, or is it primarily the result of regulatory phase-out and controlled disposal? Although the downward temporal trend is statistically well supported, the available waste management data do not indicate that large-scale mineralogical transformation technologies played a major role in Hungary during the study period. In principle, chrysotile asbestos can lose its fibrous structure at high temperatures, undergoing transformation into non-fibrous silicate phases such as forsterite or enstatite [27,28]. Under appropriate thermal conditions, this process could provide genuine detoxification. However, ACFMs are composite products that also contain binders, fillers, and metallic additives. Their thermal treatment is therefore technologically demanding and may generate secondary emissions, including particulate matter and gaseous combustion products, if process conditions are not carefully controlled. High temperature alone does not guarantee complete detoxification, particularly if combustion is incomplete or mineral conversion is partial.

The empirical pattern observed in Hungary is more consistent with containment and progressive stock depletion than with widespread thermal destruction. The dominant features of the dataset are the pre-ban disposal peak, the sharp collapse after 2005, and the subsequent gradual decline. This sequence is more readily explained by regulatory prohibition, centralized hazardous waste handling, and the slow exhaustion of legacy materials than by the deployment of advanced treatment technologies. The persistence of treatment activity, together with strong regional centralization, further supports the interpretation that asbestos has mainly been isolated and managed rather than systematically transformed. This distinction is important from a public health perspective. Landfill containment and controlled disposal reduce exposure by limiting contact with hazardous material, but they do not eliminate asbestos itself. The post-ban decline should therefore be understood as a reduction in circulation, not as definitive eradication from the technical system. In this sense, the Hungarian asbestos legacy persists in stabilized form within the waste management infrastructure, and continued control of handling, transport, and disposal remains necessary.

**Figure 4:**
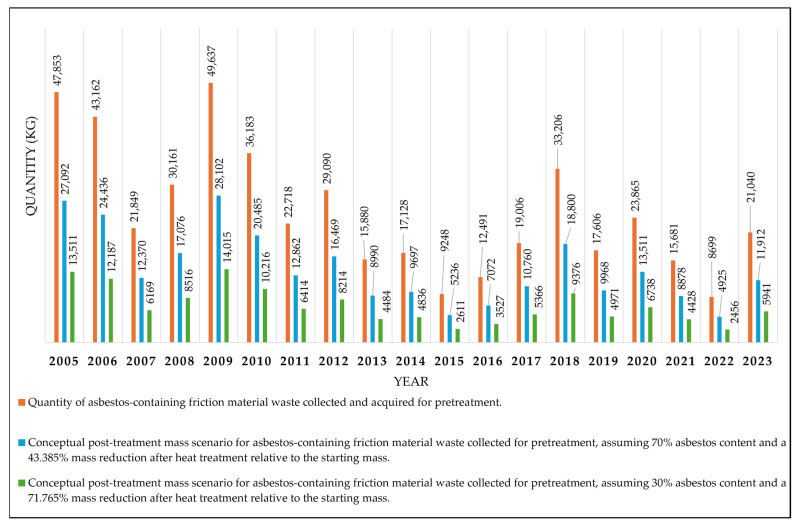
Conceptual post-treatment mass scenarios for the reduction in the mass of ACFW if they were subjected to heat treatment in Hungary, 2005–2023 (kg).

Annual quantities of ACFW collected and acquired for pretreatment in Hungary between 2005 and 2023, compared with two hypothetical post-treatment mass scenarios. The orange bars show the original collected quantity, while the blue and green bars represent estimated residual masses after heat treatment under assumed asbestos contents of 70% and 30%, respectively. Figure 4 is intended to illustrate the potential magnitude of mass reduction under different compositional assumptions and does not represent directly observed treatment outputs. Accordingly, Figure 4 should be interpreted as a theoretical visualization of possible mass-reduction scenarios, not as empirical evidence of thermal treatment performance in Hungary.

## 4. Discussion

The present analysis demonstrates that nearly two decades after the national asbestos ban entered into force on 1 January 2005, ACFMs remain detectably present in the Hungarian hazardous waste stream. Beyond confirming a declining temporal pattern, the key implication of the results is that regulatory prohibition does not automatically remove hazardous materials already embedded in durable technical systems. The regulatory shock associated with the introduction of the ban is clearly visible in the dataset. The sharp reduction after the pre-ban disposal peak indicates that the prohibition substantially reduced the circulation of new ACFMs; however, the continued post-ban occurrence of reported quantities points to the slow release of legacy components from vehicles, machinery, and storage systems.

This interpretation shifts the focus from simple trend description to the long-term governance problem created by embedded hazardous materials. The post-2005 trajectory does not exhibit complete disappearance but rather gradual reduction. Accordingly, the asbestos legacy should be understood as a delayed material-flow issue, in which legally banned components continue to enter the waste system through maintenance, dismantling, and disposal processes. The spatial dimension of the results reveals even more significant structural implications. The observed difference between regions of generation and regions of treatment suggests that asbestos-related occupational and environmental burdens are not necessarily located where the waste originally arises. Instead, hazardous waste logistics redistribute risk toward specialized collection and pre-treatment nodes. Such spatial decoupling implies that exposure risk is not evenly distributed across the national territory but may be intensified in regions hosting centralized facilities.

The sectoral distribution further clarifies the structural embedding of asbestos legacy. Rather than indicating a generalized economy-wide waste problem, the concentration of ACFW in automotive-related activities shows that residual asbestos circulation remains closely linked to the vehicle life cycle. The predominance of maintenance and repair activities suggests that contemporary exposure risk is no longer associated with primary production but with secondary handling processes, including dismantling, servicing, and waste preparation. This finding is important because it indicates a shift in the asbestos risk profile from manufacturing exposure toward downstream occupational exposure in maintenance and waste-management environments. The persistent surplus of collected over generated quantities further indicates that the Hungarian system continues to process historical stockpiles rather than only contemporaneously generated waste. The hazardous waste stream behaves as a delayed-release system in which legacy components embedded in technical infrastructure gradually enter the disposal chain. This stock-and-flow dynamic helps explain why measurable quantities can remain present long after regulatory prohibition, without implying renewed asbestos use. The decline observed in the dataset is therefore more accurately interpreted as progressive depletion of embedded legacy materials rather than immediate elimination.

The absence of evidence for widespread mineralogical transformation through high-temperature treatment supports this interpretation. Although thermal treatment can transform chrysotile under appropriate high-temperature conditions, this should not be equated with evidence that such treatment was systematically applied to ACFW in Hungary during the study period. Chrysotile fibers may lose their original fibrous structure and transform into non-fibrous silicate phases, including forsterite and enstatite, under suitable thermal conditions [27,28]. However, the available administrative waste data do not provide direct information on mineralogical conversion, post-treatment residue composition, or actual thermal treatment efficiency. Consequently, the observed decline in waste volumes appears to be driven primarily by regulatory phase-out, fleet renewal, and controlled disposal rather than documented physicochemical destruction. The asbestos legacy is therefore more appropriately interpreted as being stabilized and progressively managed, rather than definitively eradicated from the technical system. Long-term management obligations persist within the hazardous waste infrastructure.

From a public health perspective, the findings underscore that asbestos risk in the post-ban era has not disappeared but has shifted in character. Exposure risk is no longer production-based but is embedded in maintenance, dismantling, transport, and waste-processing chains. The potentially exposed working population therefore includes vehicle dismantling workers, automotive maintenance personnel, hazardous waste handlers, transport and storage workers, recycling operators, and landfill personnel involved in the handling or disposal of asbestos-containing friction components. At the same time, the administrative dataset does not allow a direct estimate of the number of exposed workers, meaning that the results identify exposure contexts rather than quantify individual occupational exposure. The centralization of treatment activities implies localized occupational risk concentration, even as national quantities decline. Therefore, continued monitoring of hazardous waste facilities, strict enforcement of occupational safety protocols, and targeted regional surveillance remain essential. The Hungarian case illustrates that asbestos prohibition effectively halted new material inflows but did not eliminate the long-term legacy embedded in durable mechanical systems. The persistence of measurable waste flows, the structural centralization of treatment, and the economic concentration within automotive sectors collectively demonstrate that asbestos management in the post-ban era remains an active environmental and occupational health challenge rather than a resolved historical issue.

## 5. Conclusions

This study demonstrates that ACFMs remain measurably present in the Hungarian hazardous waste system nearly two decades after the national asbestos ban entered into force. Although the reported quantities show a statistically significant long-term decline, their continued occurrence confirms that regulatory prohibition alone does not eliminate hazardous materials already embedded in long-life technical systems. The dataset reflects a strong regulatory shock around the introduction of the ban, followed by a prolonged post-ban phase in which residual asbestos-containing components continue to enter the waste stream through gradual fleet renewal, dismantling, maintenance, and stock depletion rather than through renewed asbestos use.

A key structural finding is the territorial centralization of waste treatment, particularly in Northern Hungary, where collected and pre-treated quantities substantially exceed locally generated amounts. This demonstrates that asbestos-related environmental burden and occupational exposure risk are geographically redistributed through centralized hazardous waste infrastructure. Sectoral analysis further reveals that ACFW is concentrated within automotive-related economic activities, confirming that the asbestos legacy is closely linked to vehicle lifecycle management. In the post-ban era, asbestos risk has therefore shifted from primary production to maintenance, dismantling, transport, and waste-processing environments.

The persistent surplus of collected over generated quantities suggests that the Hungarian system continues to process historical stockpiles and delayed material flows. The observed decline should therefore be interpreted as progressive management and depletion of embedded legacy materials, rather than as evidence of systematic mineralogical destruction. While high-temperature treatment could theoretically eliminate asbestos fiber morphology under appropriate technical conditions, the available administrative waste data do not provide direct evidence that such transformation was widely applied during the study period. The results therefore identify exposure contexts and material-flow patterns rather than directly measuring worker exposure or estimating the number of exposed workers.

From an environmental public health perspective, the findings emphasize that asbestos management remains an active, long-term challenge. Even in the absence of new production, durable technical infrastructures generate delayed hazardous flows for decades. Effective policy responses should therefore prioritize continued occupational monitoring in dismantling and treatment facilities, region-specific risk management in centralized processing areas, and long-term surveillance of disposal sites. The Hungarian case illustrates a broader principle relevant to hazardous material governance: material bans interrupt new inflows but do not dissolve legacy stocks embedded in socio-technical systems. Consequently, environmental and occupational health strategies must extend beyond prohibition and address the structural and temporal persistence of hazardous material residues within complex industrial economies.

## Data Availability

No new data were created in this study. The data analyzed in this study are available from [https://web.okir.hu/sse/?group=EHIR] (accessed on 1 November 2025) under the relevant access conditions.

## Abbreviations

The following abbreviations are used in this manuscript:

ACFMs	asbestos-containing friction materials
ACFW	asbestos-containing friction waste
EWC	European Waste Catalogue
